# Fluorescence Sensors Based on Hydroxycarbazole for the Determination of Neurodegeneration-Related Halide Anions

**DOI:** 10.3390/bios12030175

**Published:** 2022-03-14

**Authors:** Víctor González-Ruiz, Ángel Cores, M. Mar Caja, Vellaisamy Sridharan, Mercedes Villacampa, M. Antonia Martín, Ana I. Olives, J. Carlos Menéndez

**Affiliations:** 1 Unidad de Química Analítica, Departamento de Química en Ciencias Farmacéuticas, Facultad de Farmacia, Universidad Complutense de Madrid, 28040 Madrid, Spain; victor.gonzalez@unige.ch (V.G.-R.); mcaja01@ucm.es (M.M.C.); mantonia@ucm.es (M.A.M.); 2 School of Pharmaceutical Sciences, Institute of Pharmaceutical Sciences of Western Switzerland, University of Geneva, Rue Michel-Servet 1, 1211 Geneva 4, Switzerland; 3 Swiss Centre for Applied Human Toxicology (SCATH), 4055 Basel, Switzerland; 4 Unidad de Química Orgánica y Farmacéutica, Departamento de Química en Ciencias Farmacéuticas, Facultad de Farmacia, Universidad Complutense de Madrid, 28040 Madrid, Spain; acores@ucm.es (Á.C.); sridharan.che@cujammu.ac.in (V.S.); mvsanz@ucm.es (M.V.); 5 Department of Chemistry and Chemical Sciences, Central University of Jammu, Rahya-Suchani (Bagla), District-Samba, Jammu 181143, J&K, India

**Keywords:** fluorescent sensors, hydrogen bonds, carbazole derivatives, microwave-assisted synthesis, halide toxicity, halide quantitation

## Abstract

The environmental presence of anions of natural origin or anthropogenic origin is gradually increasing. As a tool to tackle this problem, carbazole derivatives are an attractive gateway to the development of luminescent chemosensors. Considering the different mechanisms proposed for anion recognition, the fluorescence properties and anion-binding response of several newly synthesised carbazole derivatives were studied. Potential anion sensors were designed so that they combined the native fluorescence of carbazole with the presence of hydrogen bonding donor groups in critical positions for anion recognition. These compounds were synthesised by a feasible and non-expensive procedure using palladium-promoted cyclodehydrogenation of suitable diarylamine under microwave irradiation. In comparison to the other carbazole derivatives studied, 1-hydroxycarbazole proved to be useful as a fluorescent sensor for anions, as it was able to sensitively recognise fluoride and chloride anions by establishing hydrogen bond interactions through the hydrogen atoms on the pyrrolic nitrogen and the hydroxy group. Solvent effects and excited-state proton transfer (ESPT) of the carbazole derivatives are described to discard the role of the anions as Brönsted bases on the observed fluorescence behaviour of the sensors. The anion–sensor interaction was confirmed by ^1^H-NMR. Molecular modelling was employed to propose a mode of recognition of the sensor in terms of complex stability and interatomic distances. 1-hydroxycarbazole was employed for the quantitation of fluoride and chloride anions in commercially available medicinal spring water and mouthwash samples.

## 1. Introduction

Anions occur naturally in many mammalian cells and are involved in numerous physiological and biochemical homeostasis processes. Thus, the fluoride anion, in addition to acting as an oxidant, is capable of crossing the blood–brain barrier and it not only causes neuronal damage but also has negative effects on certain enzymes such as cholinesterases. The importance of certain halides in the production of ROS has recently been highlighted. The activity of myeloperoxidase during cellular respiration generates hypochlorous acid from chloride anions [1,2] and constitutes a source of free radical production and oxidative stress. It is well known that several diseases of the nervous system are closely connected with neuroinflammatory processes (Alzheimer’s disease, Parkinson’s disease, amyotrophic lateral sclerosis) and occur with a clear overproduction of ROS and RNOS [3,4]. Thus, anions are associated with important damages in vulnerable proteins involved in neurodegenerative diseases [5,6].

Dysregulation of chloride anion levels is related with neurodegenerative diseases, thus it is decreased in the case of amyotrophic lateral sclerosis [7] and it is increased in the case of Parkinson’s disease [8].

The presence of chloride anion in water in millimolar concentration range is relevant because of its role in the oxidation and degradation of organic molecules [9]. The control of chloride anion in drinking water is a mandatory analytical determination in the official methods of analysis. Therefore, the determination of this anion by means of fluorescent sensors is an alternative to the official methods of the water quality control agencies.

Beyond neurodegeneration, the fluoride anion has considerable toxicological importance due to its ability to induce alterations of synapsis-related proteins [10] and, various abnormalities in blood cells [11], among other toxic effects [12,13,14,15,16,17]. Fluorine is one of the most abundant elements in the earth’s crust and so the fluoride anion is easily accumulated in the environment. The presence of fluoride in drinking waters involves dementia risk [18] and causes oxidative stress due to decreased levels of reduced glutathione, an effect that has also been associated with chloride anions [19].

Due to the presence of anions in the environment and their significant impact on health, analytical tools are needed to detect them. Fluorescent sensors are attractive for this purpose due to the excellent sensitivity of fluorescence as an analytical technique and the existence of a variety of events such as excited-state proton transfer (ESPT) [20,21,22,23], photoinduced electron transfer (PET), inter- or intramolecular charge transfer (ICT) or fluorescence resonance energy transfer (FRET) that can be successfully exploited for developing sensing compounds with satisfactory analytical features [24,25]. An elegant alternative for anion recognition consists of the interaction of the amino acid carboxylate group of tryptophan and phenylalanine with metals that have previously formed complexes (chelates) with functionalised tris(2-aminoethyl)amine linked to fluorescent moieties (anthracene, naphthalene...) [26]. Anion sensing is an active research field in supramolecular chemistry involving transition metal ions capable of forming stable complexes in water [27,28]. Moreover, anion sensing generally involves molecular recognition of analytes via hydrogen bonding or hydrophobic interactions [29,30,31,32,33]. Due to the environmental and toxicological significance of the fluoride anion, its sensors have been reviewed in the literature [34,35].

Carbazole derivatives are a smart gateway to the development of luminescent chemosensors, not only due to their native fluorescence but also because of their chemical stability and the relative easiness with which functionalised derivatives can be synthesised. The carbazole moiety presents particular photophysical, excited proton transfer and hydrogen bonding properties; thus, the acidity of nitrogen is higher in the excited state than in the ground state, and it is able to establish hydrogen bonds related to energy transfer processes [36]. Other fluorescence sensors are based on fluorescence quenching phenomena, carbazole quenching is dependent on temperature [37], the rate constants for excited state deprotonation reactions of carbazole are also related to temperature, and thus deactivation of protonated excited states is easier at higher temperatures [38].

This work describes the usefulness of carbazole derivatives as fluorescent “turn-on” anion sensors. The anions are molecularly recognised through interaction with both NH (carbazole) and OH (phenolic) groups, being important in the formation of hydrogen/halogen bonds. The distance between the NH and OH groups is crucial for the charge transfer process to be effective in enhancing fluorescence.

## 2. Materials and Methods

### 2.1. General Experimental Information

The carbazole derivatives were characterised by NMR spectra obtained on a Bruker Avance 250 spectrometer working at 250 MHz for ^1^H and 63 MHz for ^13^C and operated via the standard Bruker software (CAI de Resonancia Magnética Nuclear, Universidad Complutense). Infrared spectra were recorded on a Perkin Elmer Paragon 1000 FT-IR spectrophotometer, with all compounds examined as KBr pellets or as thin films on NaCl 20 disks. Melting points were measured on a Reichert 723 hot stage microscope, and are uncorrected. Elemental analyses were performed by the CAI de Microanálisis Elemental, Universidad Complutense, using a Leco 932 CHNS combustion microanalyser.

Ultraviolet–visible absorption spectra were obtained with an automatic double beam Kontron Uvikon 810 spectrophotometer. Uncorrected and corrected excitation and emission spectra and measurements at fixed wavelengths were obtained with a Horiba-Jobin Yvon FluoroMax-4 spectrofluorometer equipped with the control and acquisition data software FluorEssence 2.1. Excitation and emission slits were set at 5 nm. In all experiments, quartz cells with a 1 cm path length were employed.

Carbazole (**2a**) was obtained from Merck and 2-hydroxycarbazole (**2e**) was purchased from Merck and Sigma-Aldrich, respectively. Anions, as tetrabutylammonium salts (TBA), were acquired from Sigma-Aldrich. Solvents of analytical or spectroscopic quality were acquired from SDS and Panreac, and they were used without further purification, and ultrapure water was obtained from a Milli-Q Direct 8 system (Millipore).

### 2.2. Synthesis

2-Methoxy-*N*-phenylbenzenamine (**1**): To a well-stirred solution of *o*-anisidine (10 mmol) and Cu(OAc)_2_ (1 mmol) in dry CH_2_Cl_2_ (100 mL) at room temperature under an argon atmosphere was added phenyllead triacetate (11 mmol). The mixture was stirred for 2 h, filtered through celite, and the solvent was evaporated under reduced pressure. Pure product **1** was obtained by silica gel column chromatography, using CH_2_Cl_2_/hexane (1:1 mixture) as eluent.

Pale brown liquid, yield 78%. IR (neat) 3409.5, 3046.9, 1591.9, 1516.9, 1245.5, 1115.8 cm^−1^. ^1^H NMR (CDCl_3_, 250 MHz, 25 °C) δ = 3.99 (s, 3 H), 6.31 (br s, 1 H), 6.99–7.12 (m, 4 H), 7.26–7.31 (m, 2 H), 7.38–7.49 (m, 3 H). ^13^C NMR (CDCl_3_, 63 MHz, 25 °C) δ = 56.1, 111.0, 115.2, 119.1, 120.5, 121.3, 121.7, 129.8, 133.5, 143.2, 148.8. C_13_H_13_NO (199.10): calcd. C 78.36, H 6.58, N 7.03; found C 78.03, H 6.45, N 7.00. For an alternative preparation, see [39].

1-Methoxy-9*H*-carbazole (**2b**): A mixture of **1** (10 mmol) and Pd(OAc)_2_ (10 mmol) in AcOH (40 mL) was refluxed for 3 h. The solvent was evaporated under reduced pressure, and purification of the residue by silica gel column chromatography using petroleum ether/ethyl acetate (9:1 mixture) as eluent afforded compound **2b**. Pale grey solid, yield 80%, mp 70–71 °C (65–67 °C). IR (neat) 3418.0, 3057.9, 1579.4, 1455.8, 1258.2, 1097.9 cm^−1^. ^1^H NMR (CDCl_3_, 250 MHz, 25 °C) δ = 3.83 (s, 3 H), 6.74 (dd, *J* = 7.8, 0.6 Hz, 1 H), 7.02 (d, *J* = 7.8 Hz, 1 H), 7.04–7.13 (m, 1 H), 7.24–7.28 (m, 2 H), 7.56 (d, *J* = 7.8 Hz, 1 H), 7.93 (dd, *J* = 7.8, 0.6 Hz, 1 H), 8.12 (br s, 1 H). ^13^C NMR (CDCl_3_, 63 MHz, 25 °C) δ = 55.9, 106.4, 111.5, 113.4, 119.9, 120.3, 121.1, 124.1, 124.8, 126.2, 130.3, 139.7, 146.2. C_13_H_11_NO (197.08): calcd. C 79.16, H 5.62, N 7.10; found C 78.92, H 5.55, N 7.04. For an alternative preparation, see reference [40].

1-Hydroxy-9*H*-carbazole (**2c**): To a stirred solution of **2b** (1.5 mmol) in dry CH_2_Cl_2_ (20 mL) was slowly added a solution of BBr_3_ (3 mmol) in dry CH_2_Cl_2_ (10 mL). The mixture was stirred at room temperature for 2 h. After completion of the reaction, the excess reagent was destroyed carefully by adding cold water (25 mL). The organic layer was separated, washed twice with water, dried with anhydrous Na_2_SO_4_, and the solvent was evaporated. The analytically pure product **2c** was obtained through silica column chromatography, using petroleum ether/ethyl acetate (9:1 mixture) as eluent. Pale grey solid, yield 98%, mp 164–165 °C (165–167 °C). IR (KBr) 3432.1, 3221.6, 3048.8, 1581.9, 1503.9, 1253.5 cm^−1^. ^1^H NMR (DMSO-d_6_, 250 MHz, 25 °C) δ = 6.87 (dd, *J* = 7.6, 0.9 Hz, 1 H), 6.99 (t, *J* = 7.6 Hz, 1 H), 7.13 (dt, *J* = 7.8, 0.9 Hz, 1 H), 7.37 (dt, *J* = 7.8, 0.9 Hz, 1 H), 7.52 (d, *J* = 7.8 Hz, 1 H), 7.58 (d, *J* = 7.6 Hz, 1 H), 8.03 (d, *J* = 7.8 Hz, 1 H) 9.82 (s, 1 H). ^13^C NMR (CDCl_3_, 63 MHz, 25 °C) δ = 110.4, 111.5, 111.6, 118.6, 119.5, 120.5, 123.2, 124.3, 125.5, 129.8, 139.9, 143.6. C_12_H_9_NO (183.07): calcd. C 78.67, H 4.95, N 7.65; found C 78.55, H 4.88, N 7.61. For an alternative preparation, see [41].

### 2.3. Spectrofluorimetric Study of Sensors

#### 2.3.1. General Procedure

Stock solutions of **1**, **2a**–**2e** and **3** were prepared in concentration 5.0 × 10^−3^ M using ethanol as solvent. Aliquots of this solution were taken and diluted up to 5.0 × 10^−5^ M in ethanol and the UV–vis absorption spectra were obtained. In the case of carbazole (**2a**), the concentration value was corrected using the experimental value of absorbance and the reference value of molar absorptivity. For the other compounds (**1**, **2b**–**2e** and **3**), the molar absorptivities were calculated from the slopes of the corresponding calibration curves in ethanol and these values were employed to assure the value of the carbazole derivative concentration. Considering the verified concentrations of the stock solutions of **1**, **2a**–**2e** and **3**, aliquots were taken and diluted to 1.0 × 10^−5^ M in ethanol and then, fluorescence spectra were obtained.

#### 2.3.2. Solvatochromic Effect Study

Aliquots of the ethanolic stock solutions of the **2c** and **2e** were taken and the solvent was evaporated in vacuo at room temperature, resulting in thin films of carbazole derivatives left on the walls of a round bottom flask. An adequate volume of the corresponding solvent was added to obtain a concentration of 1.0 × 10^−5^ M. After 30 min under magnetic stirring the fluorescence excitation and emission spectra were recorded. This procedure was used to ensure that changes produced in the spectra or in the fluorescence intensity are only due to the environmental effects of solvents.

#### 2.3.3. Excited-State Proton Transfer Reactions in Organic and Aqueous Solvents

Additions of NaOH, Na_2_CO_3_, NaHCO_3_, NH_4_OH and triethylamine were carried out over **2c** and **2e** solutions at concentration 1.0 × 10^−5^ M in aqueous and organic solvents. After every addition, the fluorescence emission spectra were recorded.

### 2.4. Anion Sensing and Recognition

Tetrabutylammonium salts of anions (fluoride, chloride, bromide, acetate and cyanide) were freeze-dried for 48 h before use. The stock anion solutions were prepared at 1.0 mM using ethanol, acetone or acetonitrile as solvents. Working solutions of **2b**–**2e** were prepared at 1.0 × 10^−5^ M in ethanol, acetone and acetonitrile. To verify the anion sensing ability of assayed compounds **2b**–**2e**, adequate aliquots of the stock solutions of anions were added (from 0 to 0.1 mM) to the sensor solution. The anion:sensor stoichiometric ratios range from 1:10 to 2:1. The fluorescence excitation and emission spectra were recorded for each anion concentration.

For the determination of the anion–sensors affinity constants, the concentration of the sensors was fixed at 5.0 × 10^−5^ M. The anion concentrations varied from 5.0 × 10^−6^ to 1.0 × 10^−4^ M. The fluorescence intensities obtained for the different solutions allowed the calculation of the apparent association constants according to the Benessi–Hildebrand treatment [42].

### 2.5. Fluoride and Chloride Determination in Real Samples by Using 1-Hydroxycarbazole *(**2c**)* as Fluorescence Sensor

Fluoride content analysis was performed in a locally purchased commercial mouthwash (FluorKin, Kin Laboratories, Barcelona, Spain). Aliquots of 10 µL of the mouthwash were diluted up to 10 mL in acetone (sample solutions). Aliquots of 500 µL of stock solution of **2c** in ethanol were evaporated and 500 µL of the mouthwash sample solutions were added. Then, acetone was added to make a final volume of 10 mL. These solutions were spectrofluorimetrically measured. Chloride was quantified in a medicinal spring water sample (Agua de Carabaña, Carabaña, Madrid, Spain). To a 10 mL aliquot of the sample, 500 µL of stock solution of **2c** in ethanol was added. The resulting solution was evaporated using a rotary evaporator and then re-dissolved in 10 mL of acetone. After stirring, the fluorescence intensity was measured. All the determinations were performed in duplicate sets, the fluorescence intensities were interpolated in the adequate calibration curves and the final result was expressed as a percentage deviation from the value declared by the manufacturer.

### 2.6. Study of the Anion Interaction by NMR

Working solutions of the sensor and anions were prepared in d_6_-DMSO and d_6_-acetone. These compounds were mixed in stoichiometric ratios anion:sensor from 1:10 to 5:1 and the ^1^H-NMR spectra were obtained.

### 2.7. Computational Studies of the Anion Chemosensor by Molecular Modelling

Optimisation of the geometry and determination of the total energy value of the single components as well as the complexes were performed using ab initio calculations at HF/3-21G level. To compare the stability of the different complexes, the free energy was determined by the difference between the energy of the complex and the contribution of every single component. For displacement studies, the second ligand was placed in the surroundings of the optimised first ligand-complex, and then the system was again minimised. Algorithms implemented in the Spartan ’08 software (Wavefunction Inc., Irvine, CA, USA) were employed.

## 3. Results and Discussion

**Figure 1:**
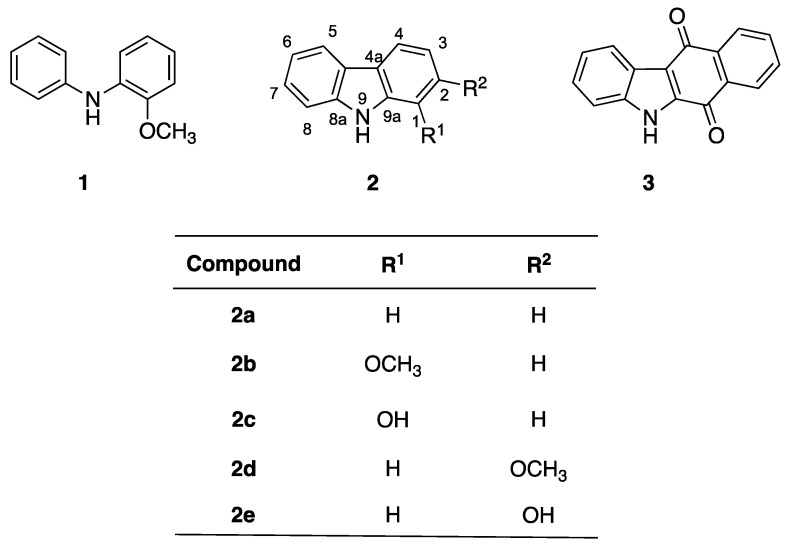
Chemical structures of the carbazole derivatives assayed as potential fluorescence sensors.

During the last decade, the design of sensors for anions has attracted much attention from researchers as a consequence of the better knowledge of anion coordination chemistry as well as the biological, toxicological and environmental relevance of anions [43,44,45]. Reversibility is an essential property of chemosensors, and anion recognition proceeds through the formation of non-covalent bonds, mainly hydrogen bonds. Therefore, amines, amides and nitrogen heterocycles are widespread in the structures of a plethora of anion chemosensors [44,45,46,47]. For this reason, we designed our compounds so that they combined native fluorescence of carbazole moiety with the presence of two functional groups with hydrogen bonding capability (NH and OH) in critical positions for anion recognition. Moreover, our compounds were also designed to evidence the relevance of the distance of the acceptor groups for anion sensing. Considering the role of the ionic radius for anions to be recognised and the potential existence of intra- or intermolecular hydrogen bonds, different derivatives of carbazole were tested, including 1-hydroxycarbazole (**2c**) and 2-hydroxycarbazole (**2e**). Furthermore, in order to check the relevance of the hydrogen bonds in the anion recognition, the behaviour of these compounds was compared with that of corresponding methoxy derivatives. Finally, quinone **3** was also assayed because its NH–oxygen distance is similar to compounds **2b** and **2c** (Figure 1), and the low fluorescence emission of quinone derivatives made it a promising sensor in the case of effective molecular recognition of the sample anions, as the base signal of the sensor could be avoided. The low native fluorescence of quinones in general, and of derivative **3** in particular, is not enhanced by interaction with halides.

### 3.1. Synthesis

**Figure 2:**
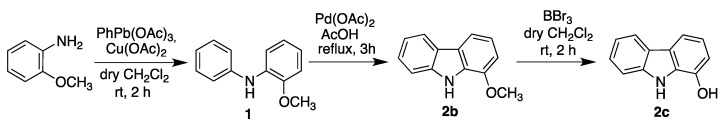
Synthesis of carbazole derivatives **2b** and **2c**.

Compound synthesis was carried out according to Figure 2. N-arylation of 2-methoxyaniline with phenyllead triacetate [48] afforded compound **1**, which was transformed into the corresponding carbazole derivative **2b** by palladium-catalysed cyclodehydrogenation. Finally, the O-demethylation of **2b** with boron tribromide afforded the hydroxyl derivative **2c**. Compounds **2a**, **2d** and **2e** were purchased from commercial sources and compound **3** was synthesised according to a literature procedure [49,50].

Compound **2c** combines two desirable features in a sensor device: quick and easily measurable response. Besides, hydroxycarbazole sensors are structurally simple compounds obtained by a feasible and inexpensive chemical synthesis.

### 3.2. Fluorescence Behaviour of Carbazole Derivatives

The significant fluorescence of the carbazole ring affords the possibility of the sensitive quantitation of analytes based on their recognition by carbazole derivatives. All compounds studied (**1**–**3**) exhibited native fluorescence, but the fluorescence intensity for compounds **1** and **3** was significantly lower than that observed for the corresponding carbazole analogues **2a**–**2e** (Table 1).

The highly rigid polycyclic systems (**2a**–**2e**) are less susceptible to the non-radiative decay process than more flexible molecules (**1**) with similar structure and length of π-electron system. For compound **3** the fluorescence intensity observed was 200 times lower than for other carbazole derivatives. Attachment of a quinone group to fluorophores is known to cause a decrease in fluorescent emission via intramolecular electron transfer from the excited fluorophore to the quinone acceptor or by transfer of excitation energy to a low-lying non-emissive charge transfer state [51].

**Figure 3:**
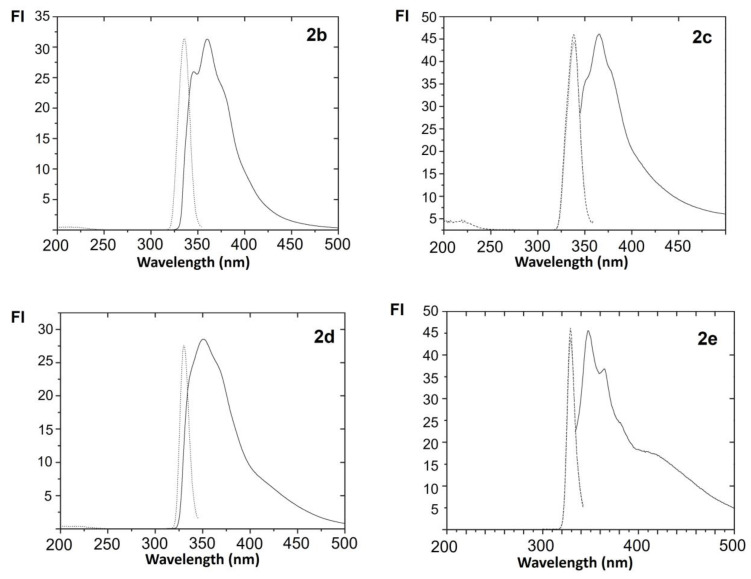
Corrected excitation and fluorescence emission spectra of **2b**–**2e** in acetone. The fluorescence intensity (FI) in the excitation and emission spectra is normalised to the same intensity value. Wavelength in nm.

The spectral shape and the fluorescence intensity are correlated to the solvent and the chemical structure of the compound studied. Figure 3 shows excitation and fluorescence emission spectra of hydroxycarbazole derivatives and methoxyxycarbazole derivatives in acetone.

The anion–sensor interactions may be influenced by the nature of solvents because fluorophores are sensitive to the microenvironment via intramolecular hydrogen bonding and thus slight changes in solvent polarity may induce remarkable solvatochromic changes in the emission properties [52]. The emission spectra of the carbazole derivatives in acetone are compared in Figure S1. Figures S2–S5 (ESI) show the excitation and emission spectra of 1-hydroxycarbazole (**2c**) and 2-hydroxycarbazole (**2e**) in ethanol and cyclohexane. In the first case, corresponding to a polar protic solvent, a broad emission band with a defined maximum is observed. In cyclohexane, an apolar solvent, the emission spectra are resolved, exhibiting three peaks as expected for an aromatic heterocycle. The emission maxima change according to the variation in the solvent polarity (Table S1 ESI). The fluorescence emission maxima and the intensity obtained for 2-hydroxycarbazole and 2-methoxycarbazole are in agreement with those described in previous literature [53].

### 3.3. Excited-State Proton Transfer (ESPT) Reactions of Carbazole Derivatives

Carbazole becomes more acidic in the excited state, its pK_a_* value being 10.98 [54]. Basic anions can lead to deprotonation of the sensor in aqueous media. In order to distinguish the fluorescence behaviour of carbazole as an anion chemosensor from the excited-state proton transfer equilibrium (ESPT), we have studied the changes in the fluorescence emission spectra caused by titration with different bases in aqueous and organic media. The titration of 1-hydroxycarbazole (**2c**) and 2- hydroxycarbazole (**2e**) in ethanol, acetone or aqueous solution with different bases (NaOH, Na_2_CO_3_, NaHCO_3_, NH_4_OH) quenches the fluorescence emission at 350–390 nm and causes the presence of a new emission band with a maximum centred at 430–450 nm, as can be appreciated in Figure S6A,B (ESI). The presence of this new emission band can be attributed to the carbazole anion produced in aqueous/organic solvents [55]. These experiments support that the anion interaction proceeds through hydrogen bonding with the sensor rather than by sensor deprotonation. Figure S7 corresponds to another experiment of fluoride titration of **2c** in acetone covering up to 500 nm showing the absence of fluorescence emission in the region 400–500 nm corresponding to anion emission. The fluorescence anion emission was not observed for carbazole derivatives in the presence of F^−^, Cl^−^, Br^−^, CN^−^, AcO^−^, OH^−^ as TBA salts and in absence of water.

### 3.4. Anion Sensing and Recognition

Fluorescence quenching and fluorescence enhancement are the most common luminescence phenomena exploited for analytical sensing purposes. Carbazole derivatives **2b**–**2e** were studied as sensors in acetone. In order to establish anion sensitivity for our chemosensors, the anions studied were F^−^, Cl^−^, Br^−^, CN^−^, Ac^−^ and OH^−^.

**Figure 4:**
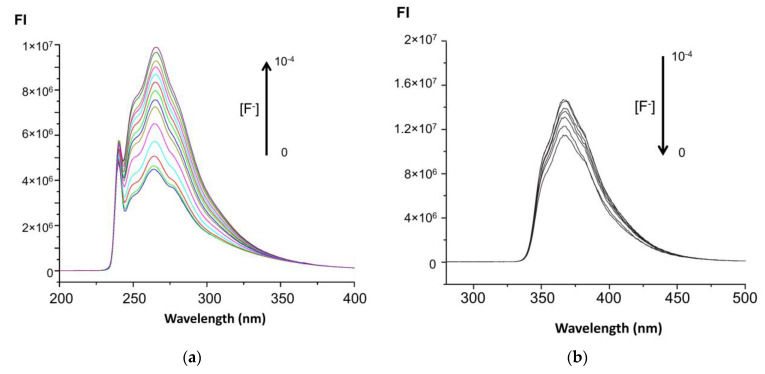
Overlaid fluorescence emission spectra of **2c** in acetone (**a**) (λ_ex_ = 340 nm) and in ethanol (**b**) (λ_ex_ = 290 nm), in the presence of increasing concentrations of fluoride anion. FI: fluorescence intensity in arbitrary units. Wavelength in nm.

The addition of increasing amounts of anions to a solution of **2c** (1-hydroxycarbazole) in acetone causes an increase in the fluorescence emission intensity of the sensor in acetone as can be appreciated in Figure 4a. Thus, these anions “turn-on” and enhance the fluorescence response of the sensor. The fluorescence enhancement factor (FI − FI_0_/FI_0_) was higher in the case of halides, and it was very small for acetate anion and intermediate for OH^−^ (Figure S8 ESI). The emission spectral shape does not change with the increasing amounts of anions. Thus, compound **2c** is proposed as a fluorescence sensor for anions. The fluorescence behaviour is in agreement with the changes observed in the signals corresponding to -NH and -OH detected by ^1^H-NMR in the presence of anions, as described below.

For carbazole derivatives **2b**, **2d** and **2e**, studied as sensors in acetone, the presence of increasing amounts of anions caused a decrease in the fluorescence emission and fluorescence enhancement factor (Figures S9 and S10 ESI) as a consequence of the dilution effect. Besides, neither spectral changes nor fluorescence emission increases were observed for compound **3** in the presence of anions in acetone. A decrease in the fluorescence intensity of all carbazole derivatives (**2c**–**2e**) was observed in the presence of the anions studied when the solvents employed were ethanol (Figure 4b) or acetonitrile. Thus, acetone plays a key role in the enhancement of the fluorescence emission of **2c** in the presence of anions.

The existence of intermolecular charge transfer phenomena (ICT) associated with hydrogen bond formation between the solvent (acetone) and the groups NH and OH of the sensor molecule (**2c**) can explain the spectral changes observed depending on the nature of the solvent environment since, in the presence of anions, the solvent (acetone) is displaced, modifying the hydrogen bonding pattern of **2c** and increasing negative charge in the surroundings of the aromatic moiety. Therefore, the selection of solvent is crucial for adequate sensing due to its role in establishing the hydrogen bonds and in the acid–base behaviour of anions and sensors [56,57].

**Figure 5:**
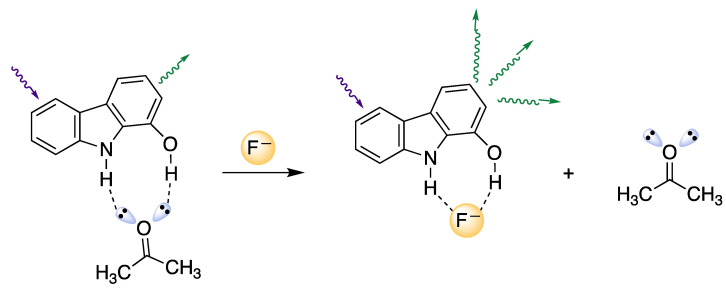
Proposed scheme for the selective sensing of a fluoride anion by compound **2c**. The increase in the fluorescence intensity of the sensor is a consequence of charge transfer and the intermolecular sensor–solvent–halide anion rearrangement.

Compound **2c** is able to establish two hydrogen bonds with the solvent (acetone), and in the presence of spherical anions with high electronic density, the anion displaces the solvent causing the above-described fluorescence enhancement (Figure 5). Consequently, the fluorescence emission is favoured and a noticeable “turn-on” fluorescence response is observed. Recognition of anions by hydrogen bonding involving NH groups in urea, amides, pyrroles and indoles has been described in the literature [58,59].

In such cases, the nitrogen atom, acting as a hydrogen donor, plays an important role to form stable adducts with anions [60]. An enhancement of the fluorescence intensity of bis-indolocarbazoles in acetone solution has been described in the literature after anion addition via a similar mechanism [61].

### 3.5. Determination of the Affinity Constants Anion–Sensor 2c

The apparent association constants (*K*_ass_) were determined by fluorescence titration in acetone. These values were deduced from double reciprocal plots [42]. For this purpose, 1/FI − FI_0_, was plotted against the reverse anion concentration, and the *K_ass_* values were calculated from the slope and ordinate values [62] as is described in the Supplementary Information. *K_ass_* values were 1.55 × 10^5^ M^−1^ and 9.64 × 10^4^ M^−1^ for fluoride and chloride, respectively, with good correlation (Table S2). Although the cyanide and acetate anions cause an increase in the fluorescence intensity, the K_ass_ values were not calculated because of their lack of fitness to the Benessi–Hildebrand model (R^2^ < 0.90). The constant values obtained for fluoride anion are similar to those described for other fluoride sensors [63,64,65].

### 3.6. Anion Recognition Studies by ^1^H-NMR

**Figure 6:**
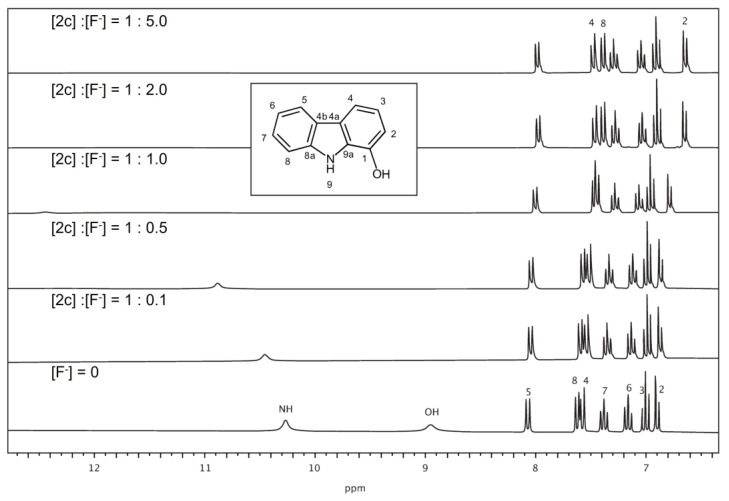
^1^H-NMR spectra of fluorescence sensor **2c** in d_6_-acetone in the presence of fluoride anions at molar ratio indicated over each spectrum.

The anion recognition ability of **2c** was also studied by ^1^H-NMR. Figure 6 and Figure S11 show the effect of increasing concentrations of the fluoride anion on the ^1^H-NMR spectrum of **2c**. The signals from OH and NH protons of **2c** are shifted downfield and become too broad for observation for the highest molar ratio of anions. These changes indicate the alteration in the electronic density of nitrogen and oxygen atoms due to the presence of anions in the surroundings of these groups. These variations in the chemical shifts are in agreement with those described in the literature [66,67,68]. The signals corresponding to the H-2 and H-8 protons, corresponding to the carbons close to the NH and OH groups, are shifted upfield with an increasing molar ratio of anions (F^−^ and OH^−^). The variation of the chemical shift of these signals in the titration with anions is shown in Figure S12. It is important to point out that although the qualitative changes seen in the ^1^H-NMR spectra are significant, the quantitative variations in the chemical shift values shown in Figure S11 are slight (0 to 0.2 ppm) for hydrogens on carbons close to the pyrrolic NH. These changes, together with the 0–2 ppm variation in the NH signal, suggest that the fluoride anion interacts efficiently to increase the rigidity of carbazole moiety, and thus enhance the fluorescence of the sensor. Significant variations (0 to 3 ppm) in the chemical shift have been described in the presence of a fluoride anion for the NH group in functionalised ureas [69]. Downfield shifts of the carbazole NH protons have been reported for other indole and carbazole derivatives acting as anion sensors [36]. Due to the rigidity imposed by the hydrogen bonds formed by the NH and OH groups of sensor **2c**, an increased fluorescence is observed upon the addition of the chloride and fluoride anions [70]. The changes observed in ^1^H-NMR spectra of **2c** in the presence of F^−^ and OH^−^ anions were quite similar, being more resolved in d_6_-DMSO with regard to d_6_-acetone (Figure 6, Figures S11 and S13).

### 3.7. Molecular Modelling Studies

Molecular modelling studies revealed that fluoride, which caused the highest increase in fluorescence intensity, was also capable of displacing acetone and forming the thermodynamically most stable complex with **2c**, as shown in Table 2, while anions that were unable to improve fluorescence intensity, such as acetate, formed higher energy complexes. These results suggest a direct relationship between the stability of the complex, association constant and fluorescence enhancement. These calculations allowed us to determine the size of the recognising cavity in **2c** by measuring the distance between NH and OH phenolic group hydrogens. The length of hydrogen bonds between these atoms and the ligands were also determined (Table S3, ESI). The results showed that fluorescence enhancement and stability of the complexes increased as the radius of the ligand decreased, suggesting that only the smallest anions could form stable hydrogen bonds avoiding steric hindrance [71]. It is remarkable that hydrogen bonding distances in the case **2c** and F^−^ were short and close to the length of a covalent bond, as previously described for other fluoride complexes [72].

**Figure 7:**
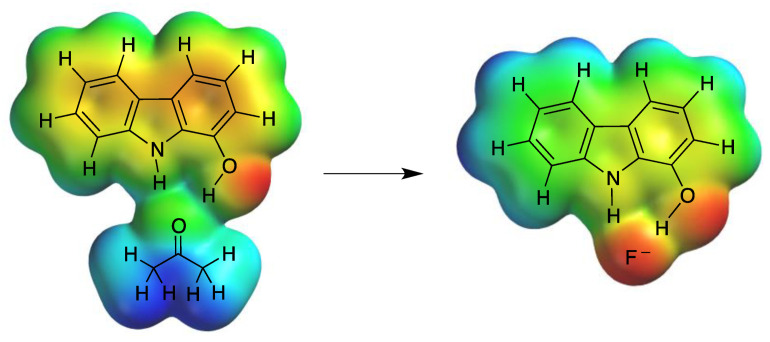
Electron density surface calculated for **2c** associated with the solvent (acetone) and fluoride.

The electron density surfaces of the sensor and the sensor–fluoride complex have also been calculated, as shown in Figure 7. It was observed that electronic density decreased around pyrrole-type and hydroxy hydrogens on **2c** as a consequence of the proximity of the strongly electronegative fluoride. The interaction of the fluoride anion with compound **2c** makes the carbazole structure more rigid and thus enhances the fluorescence emission.

### 3.8. Analytical Application of ***2c*** as “Turn-On” Fluorescence Sensor

**Figure 8:**
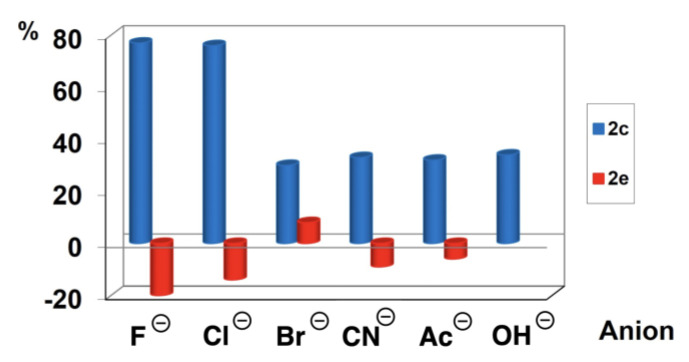
Comparison of the fluorescence response of the sensors **2c** and **2e** for the different anions studied.

While the presence of anions can be qualitatively detected by the changes in the ^1^H-NMR chemical shifts due to the alteration of the sensor–solvent hydrogen bond network after anion addition, these phenomena were quantitatively detected by fluorescence. With the aim to demonstrate the linear behaviour of the fluorescence emission intensity obtained for the sensor in the presence of increasing concentrations of anions calibration curves were performed. Compound **2c** was employed for the analysis of the different anions studied at concentrations ranging from 1.0 × 10^−5^ to 1.0 × 10^−3^ M, in the anion stock solutions. The anion concentrations in the measured solutions vary from 5.0 × 10^−6^ to 1.0 × 10^−4^ M, the concentration of the sensor in the media being constant (5.0 × 10^−5^ M). Only in the case of halides, a linear response between fluorescence intensity and anion concentration with good correlation (R^2^ > 0.93) was obtained (Table S4, ESI). On the other hand, for acetate, cyanide and hydroxide, the correlation values are non-acceptable (R^2^ < 0.87). Thus, quantitative determination of the latter anions was not possible. These results make possible the determination of chloride in medicinal spring water samples and fluoride anions in pharmaceutical samples by exploiting the fluorescence enhancement of **2c** in acetone. The agreement obtained in our experimental results and the asserted values by the manufacturer were evaluated for relative error showing a satisfactory and sensitive response of this sensor (**2c**) (Table S5, ESI). Good values of relative errors (<10%) were obtained. It is important to remark that the presence of water in the solution in a proportion higher than 5% hampers the analytical response of the sensor and, therefore, an appropriate sample pre-treatment was necessary, as described in the Experimental Section. The selectivity of sensor **2c** towards fluoride and chloride anions is due to the notable fluorescence response, as can be appreciated in Figure 8.

Although sensor **2c** shows an increase in fluorescence intensity, in acetone, in the presence of all the anions studied, the improvement in fluorescence intensity is low for the larger anions or those with lower electronegativity.

## 4. Conclusions

1-Hydroxycarbazole (**2c**) behaves as a smart and selective fluorescence chemosensor for fluoride and chloride. The role of the solvent and the NH and OH binding sites for selective interaction with anions has been discussed. The enhancement in the fluorescence emission intensity observed, in particular for fluoride and chloride anions is a consequence of specific interactions involving hydrogen bonds and an intermolecular charge transfer involving the solvent, acetone. These results are in agreement with those obtained in the ^1^H-NMR and molecular modelling experiments. The use of **2c** as a fluorescence chemosensor allows the accurate and sensitive determination of chloride and fluoride in real samples, both with an important biological significance in neurodegenerative diseases as well as due to their relevance from an environmental point of view. Compound **2c** combines two desirable features in a sensor device: quick and easily measurable response. Moreover, it is structurally simple and can be readily obtained by chemical synthesis.

## Figures and Tables

**Table 1:** Native fluorescence parameters of the compounds studied in ethanol.

Compound	λ_ex_ (nm)	λ_em_ (nm)	FI ^1^
**1**	297	352	0.22
**2a**	256, 292	338	10.0
**2b**	258, 290, 324, 336	364	62.5
**2c**	252, 290	368	71.0
**2d**	256, 302	348	42.6
**2e**	256, 302	350	100
**3**	294	326	0.04

^1^ Normalised fluorescence intensity to 100.

**Table 2:** Calculated free energy and interatomic distances.

Complex	Free Energy (kJ mol^−1^)	Difference to Acetone (kJ mol^−1^)	Ligand-NH Distance (Å)	Ligand-OH Distance (Å)
**2c**-acetone	−79.48	0.00	2.04	1.83
**2c**-F^−^	−484.67	405.19	1.54	1.21
**2c**-CN^−^	−213.29	133.81	1.93	1.65

## Data Availability

The data presented in this study are available in the article and Supplementary Materials.

## Supplementary Materials

The following supporting information can be downloaded at: https://www.mdpi.com/article/10.3390/bios12030175/s1, Figure S1: Excitation and fluorescence emission spectra of **2b**–**2e** in acetone. The fluorescence intensity (FI) in the excitation and emission spectra is normalised to the same intensity value. Wavelength in nm; Figure S2: Corrected excitation and fluorescence emission (λ_ex_ = 340 nm) spectra of **2c** in ethanol. The fluorescence intensity (FI) in the excitation and emission spectra is normalised to the same intensity value. Wavelength in nm; Figure S3: Corrected excitation and fluorescence emission (λ_ex_ = 290 nm) spectra of **2e** in ethanol. The fluorescence intensity (FI) in the excitation and emission spectra is normalised to the same intensity value. Wavelength in nm; Figure S4: Corrected excitation and fluorescence emission (λ_ex_ = 290 nm) spectra of **2c** in cyclohexane. The fluorescence intensity (FI) in the excitation and emission spectra is normalised to the same intensity value. Wavelength in nm; Figure S5: Corrected excitation and fluorescence emission (λ_ex_ = 290 nm) spectra of **2e** in cyclohexane. The fluorescence intensity (FI) in the excitation and emission spectra is normalised to the same intensity value. Wavelength in nm; Figure S6: Fluorescence emission spectra illustrating the effect of bases addition on the fluorescence of hydroxycarbazole derivatives. (A) Compound **2c**, additions of NaOH in ethanol; (A) Compound **2e**, additions of increasing amounts of Na_2_CO_3_ in water (B); Figure S7: Overlaid fluorescence emission spectra of **2c** in acetone (λ_ex_ = 340 nm), in the presence of increasing concentrations of fluoride anion. FI: Fluorescence intensity in arbitrary units. Wavelength in nm; Figure S8: Fluorescence enhancement factor (FI − FI_0_/FI_0_) obtained in the titration of compound **2c** with increasing concentrations of different anions in acetone. FI: Fluorescence emission intensity value for the examined concentration of anion. FI_0_: Fluorescence emission intensity value obtained for the sensor in absence of anion; Figure S9: Fluorescence enhancement factor (FI − FI_0_/FI_0_) obtained in the titration of compound **2c** with increasing concentrations of halide anions in ethanol. FI: Fluorescence emission intensity value for the examined concentration of anion. FI_0_: Fluorescence emission intensity value obtained for the sensor in absence of anion; Figure S10: Fluorescence enhancement factor (FI − FI_0_/FI_0_) obtained in the titration of compound **2e** with increasing concentrations of different anions in acetone. FI: Fluorescence emission intensity value for the examined concentration of anion. FI_0_: Fluorescence emission intensity value obtained for the sensor in absence of anion; Figure S11: ^1^H-NMR spectra of fluorescence sensor **2c** in d_6_-DMSO in the presence of fluoride anion at molar ratio indicated over each spectrum; Figure S12: Decrease in the ^1^H-NMR spectra of fluorescence sensor **2c** in d_6_-DMSO in the presence of fluoride anion at molar ratio indicated over each spectrum; Figure S13: ^1^H-NMR spectra of fluorescence sensor **2c** in d_6_-acetone in the presence of hydroxide anion at molar ratio indicated over each spectrum. Table S1: Solvatochromic effect. Fluorescence emission maxima and intensities (in parenthesis) of compounds **2c** and **2e** in solvents with different polarities; Table S2: Affinity constant values obtained for fluoride with sensor **2c** and chloride with sensor **2c**; Table S3: Radius predicted for the anions to be recognised by sensor **2c**; Table S4**:** Linear regression parameters obtained for the anions studied and sensor **2c** in acetone; Table S5: Determination of fluoride and chloride anions in real samples. Accuracy evaluation.
