# Supplementary material for: Fluorescence Sensors Based on Hydroxycarbazole for the Determination of Neurodegeneration-Related Halide Anions
Source: Biosensors (Basel). 2022 Mar 14;12(3):175. doi: 10.3390/bios12030175 (PMC8946780; doi:10.3390/bios12030175)
Supplement: Supplementary file 1 [file biosensors-12-00175-s001.zip › biosensors-1626546-supplementary.pdf]

Fluorescence sensors based on hydroxycarbazole for the determination of neurodegeneration-related halide anions

Víctor González-Ruiz [1,2,3], Ángel Cores [4], M. Mar Caja [1], Vellaisamy Sridharan [4,5], Mercedes Villacampa [4], M. Antonia Martín [1], Ana I. Olives [1,*] and J. Carlos Menéndez [4,*]

[1] Unidad de Química Analítica, Departamento de Química en Ciencias Farmacéuticas, Facultad de Farmacia,
Universidad Complutense de Madrid, 28040 Madrid, Spain; victor.gonzalez@unige.ch (V.G.-R.); mcaja01@ucm.es (M.M.C.); mantonia@ucm.es (M.A.M.)
[2] School of Pharmaceutical Sciences and Institute of Pharmaceutical Sciences of Western Switzerland, University of Geneva, Rue Michel-Servet 1, 1211 Geneva 4, Switzerland (V.G.-R.)
[3] Swiss Centre for Applied Human Toxicology (SCATH), 4055 Basel, Switzerland (V.G.-R.)
[4] Unidad de Química Orgánica y Farmacéutica, Departamento de Química en Ciencias Farmacéuticas, Facultad de Farmacia, Universidad Complutense de Madrid, 28040 Madrid, Spain; acores@ucm.es (Á.C.); sridharan.che@cujammu.ac.in (V.S.); mvsanz@ucm.es (M.V.)
[5] Department of Chemistry and Chemical Sciences, Central University of Jammu, Rahya-Suchani (Bagla), District-Samba, Jammu-181143, J&K, India; sridharan.che@cujammu.ac.in (V.S.)
***** Correspondence: aiolives@ucm.es (A.I.O.); josecm@ucm.es (J.C.M.)

Figures S1 to S13
Tables S1 to S5

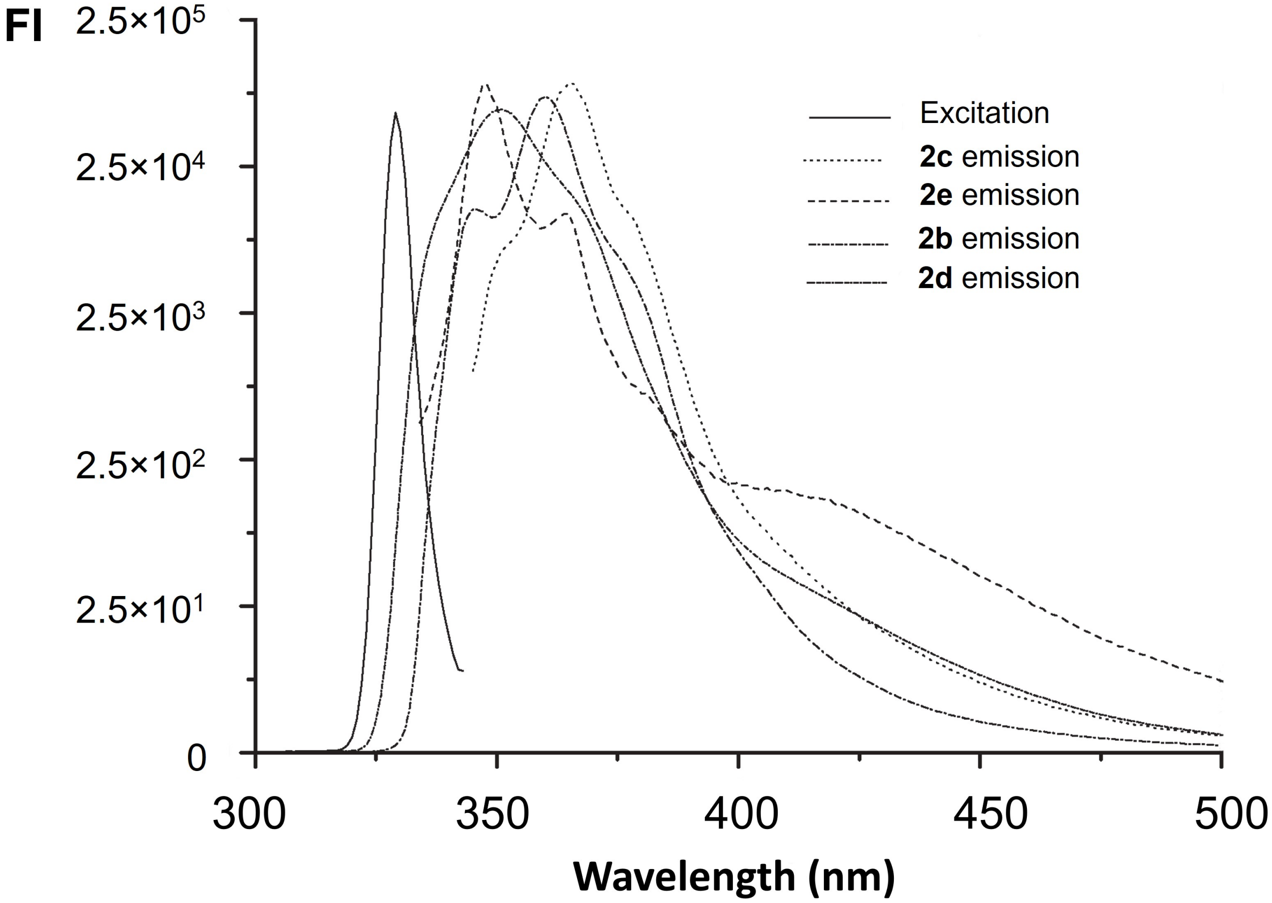

Figure S1. Excitation and fluorescence emission spectra of **2b**-**2e** in acetone. The fluorescence intensity (FI) in the excitation and emission spectra is normalized to the same intensity value. Wavelength in nm.

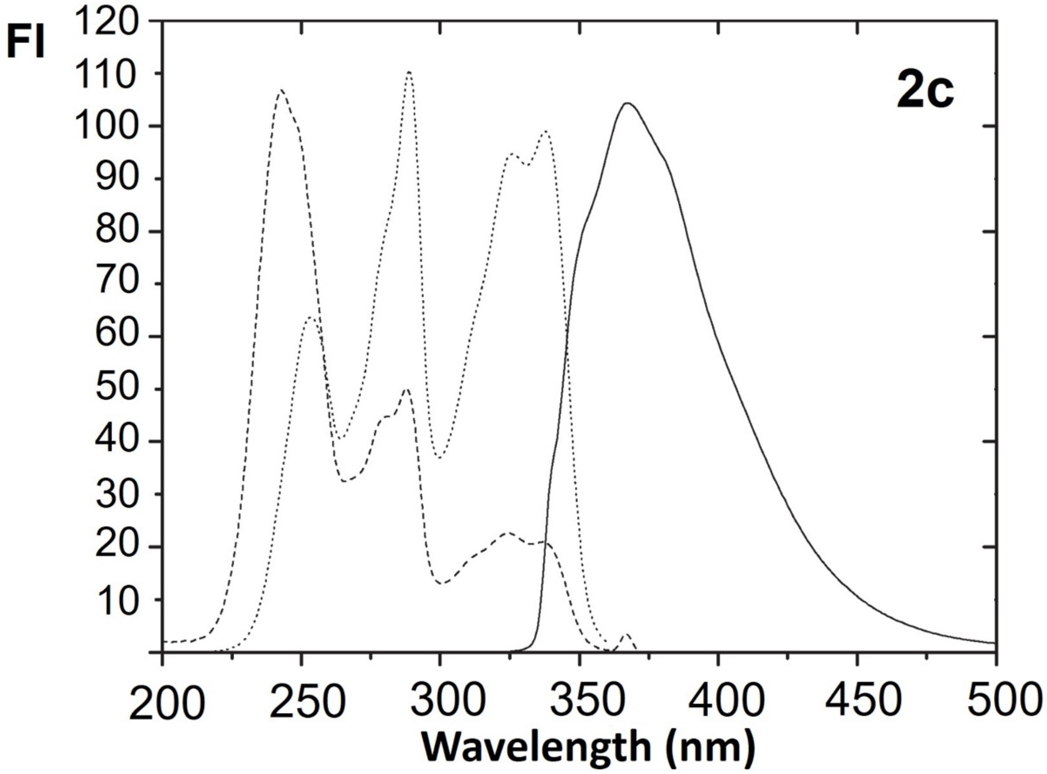

Figure S2. Corrected excitation and fluorescence emission (λ_{ex} = 340 nm) spectra of **2c** in ethanol. The fluorescence intensity (FI) in the excitation and emission spectra is normalized to the same intensity value. Wavelength in nm.

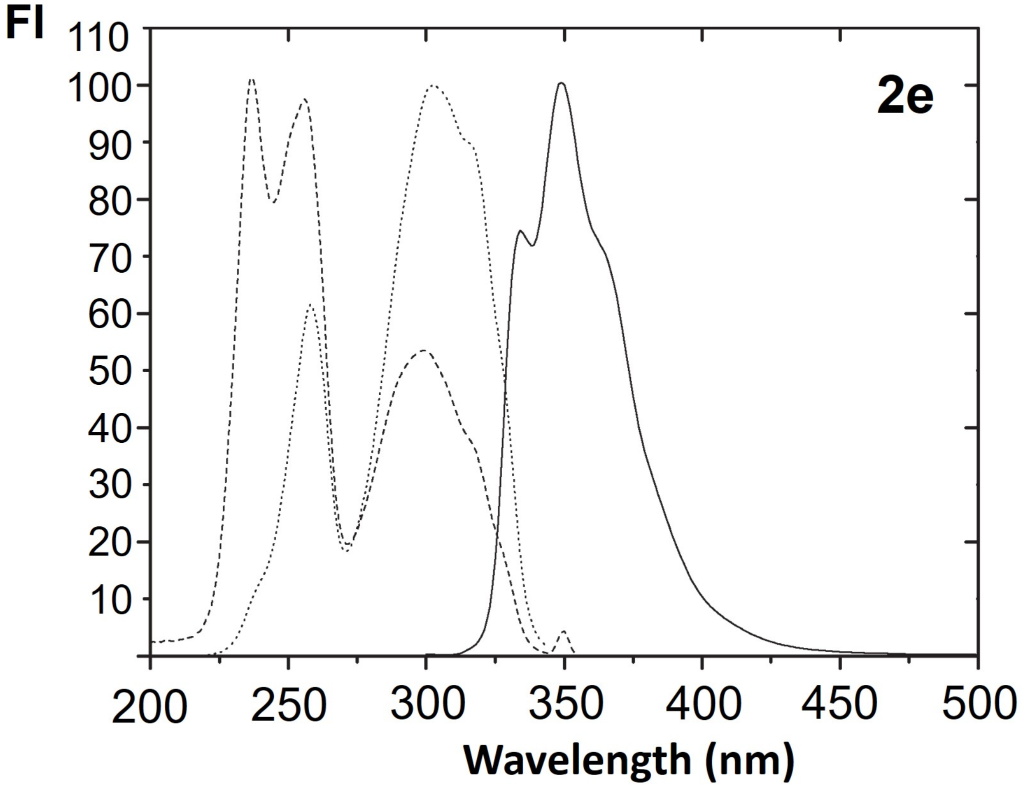

Figure S3. Corrected excitation and fluorescence emission (λ_{ex} = 290 nm) spectra of **2e** in ethanol. The fluorescence intensity (FI) in the excitation and emission spectra is normalized to the same intensity value. Wavelength in nm.

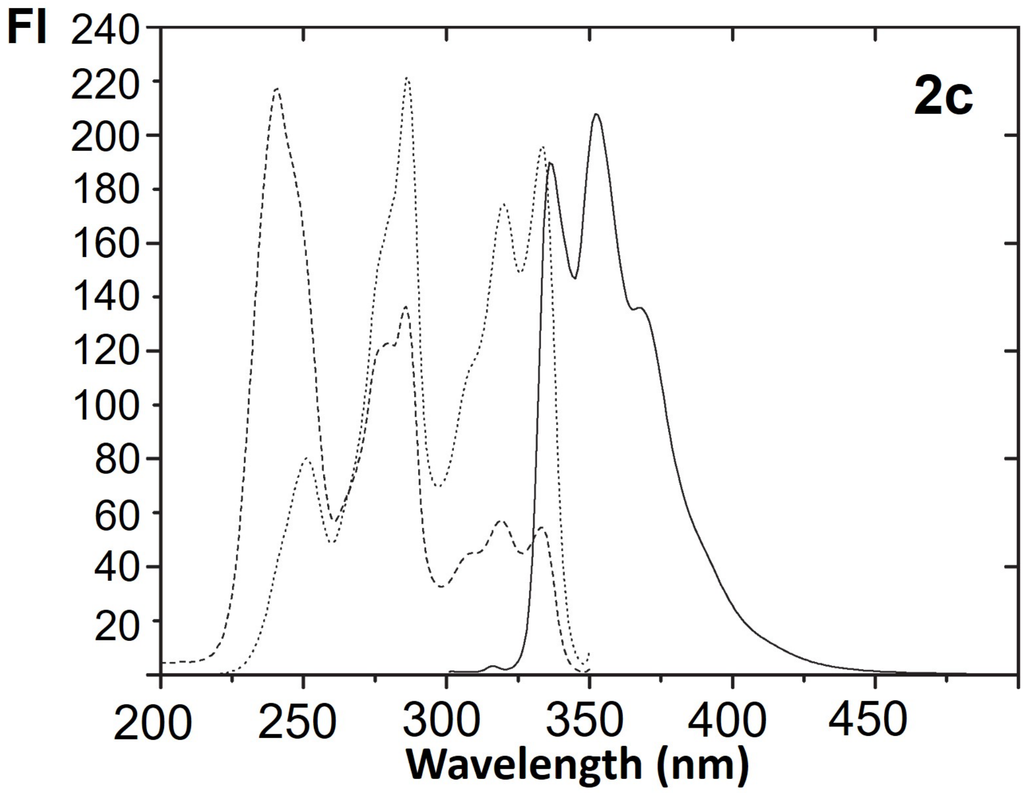

Figure S4. Corrected excitation and fluorescence emission (λ_{ex} = 290 nm) spectra of **2c** in cyclohexane. The fluorescence intensity (FI) in the excitation and emission spectra is normalized to the same intensity value. Wavelength in nm.

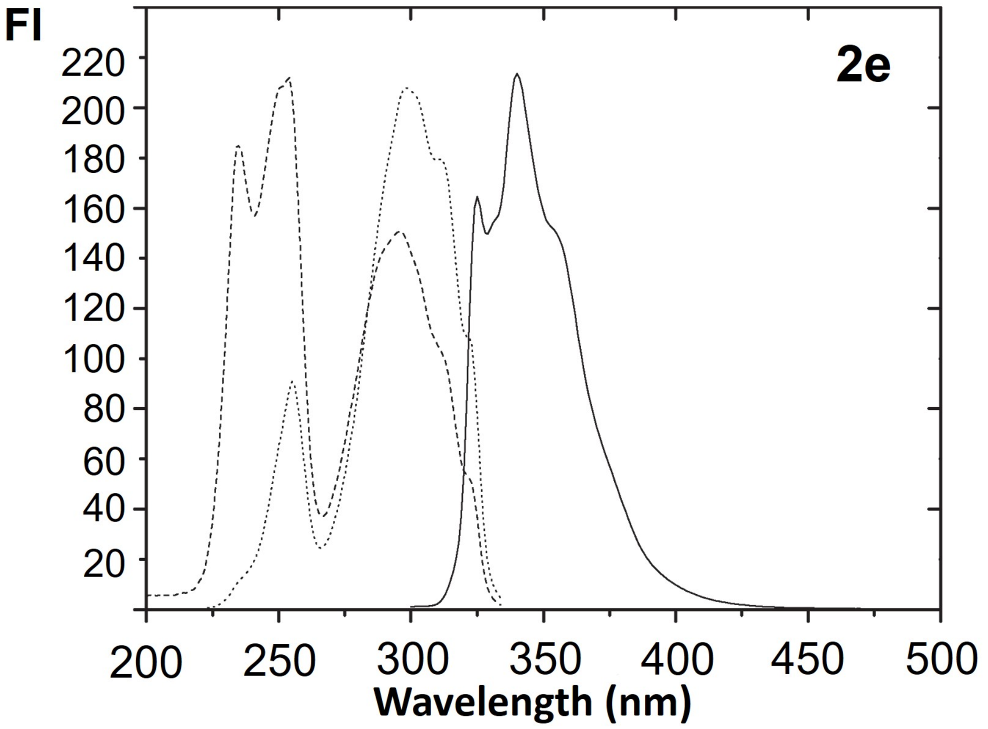

Figure S5. Corrected excitation and fluorescence emission (λ_{ex} = 290 nm) spectra of **2e** in cyclohexane. The fluorescence intensity (FI) in the excitation and emission spectra is normalized to the same intensity value. Wavelength in nm.

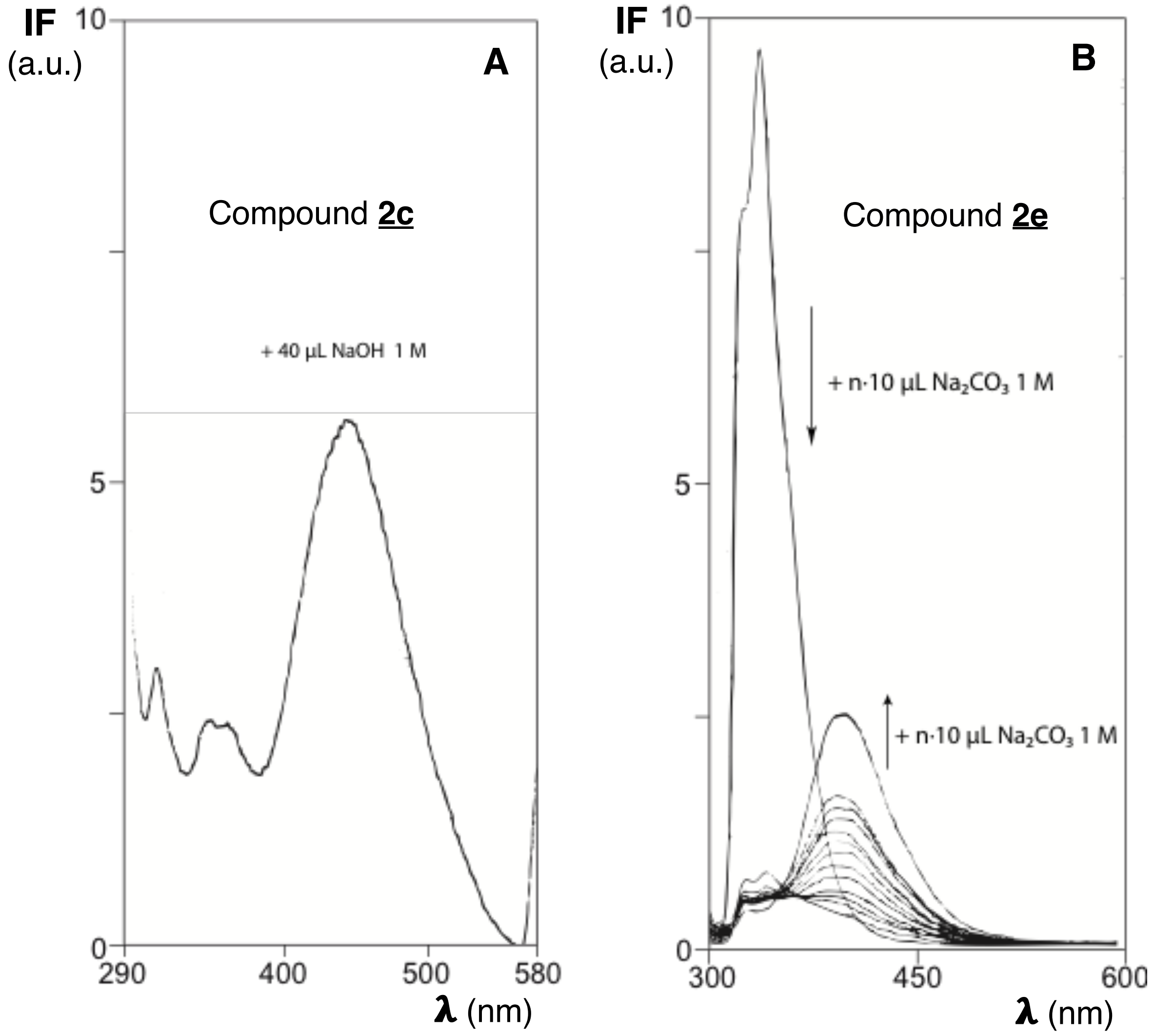

Figure S6. Fluorescence emission spectra illustrating the effect of bases addition on the fluorescence of hydroxycarbazole derivatives. (A) Compound **2c**, additions of NaOH in ethanol; (A) Compound **2e**, additions of increasing amounts of Na_2CO_3 in water (B).

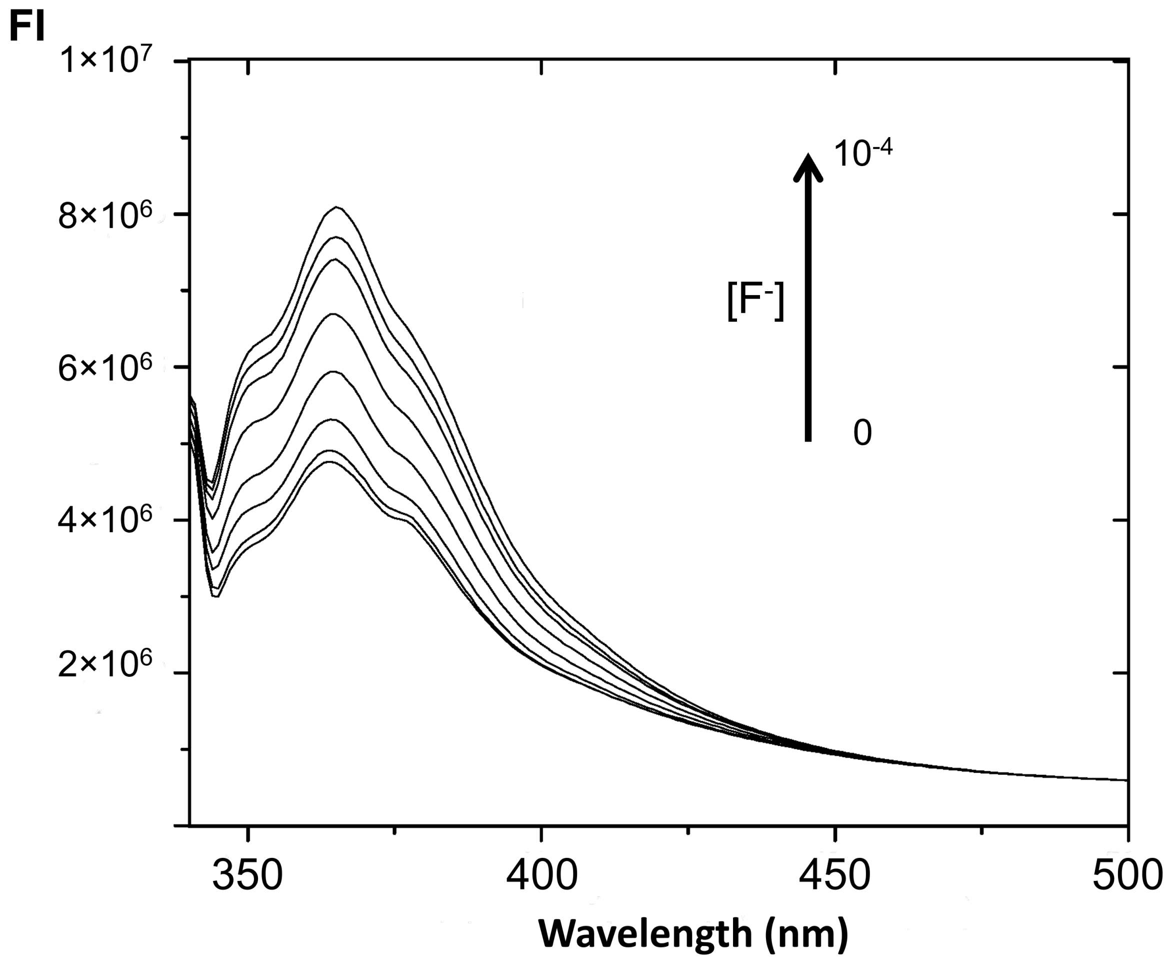

Figure S7. Overlaid fluorescence emission spectra of **2c** in acetone (λ_{ex}= 340 nm), in the presence of increasing concentrations of fluoride anion. FI: Fluorescence intensity in arbitrary units. Wavelength in nm.

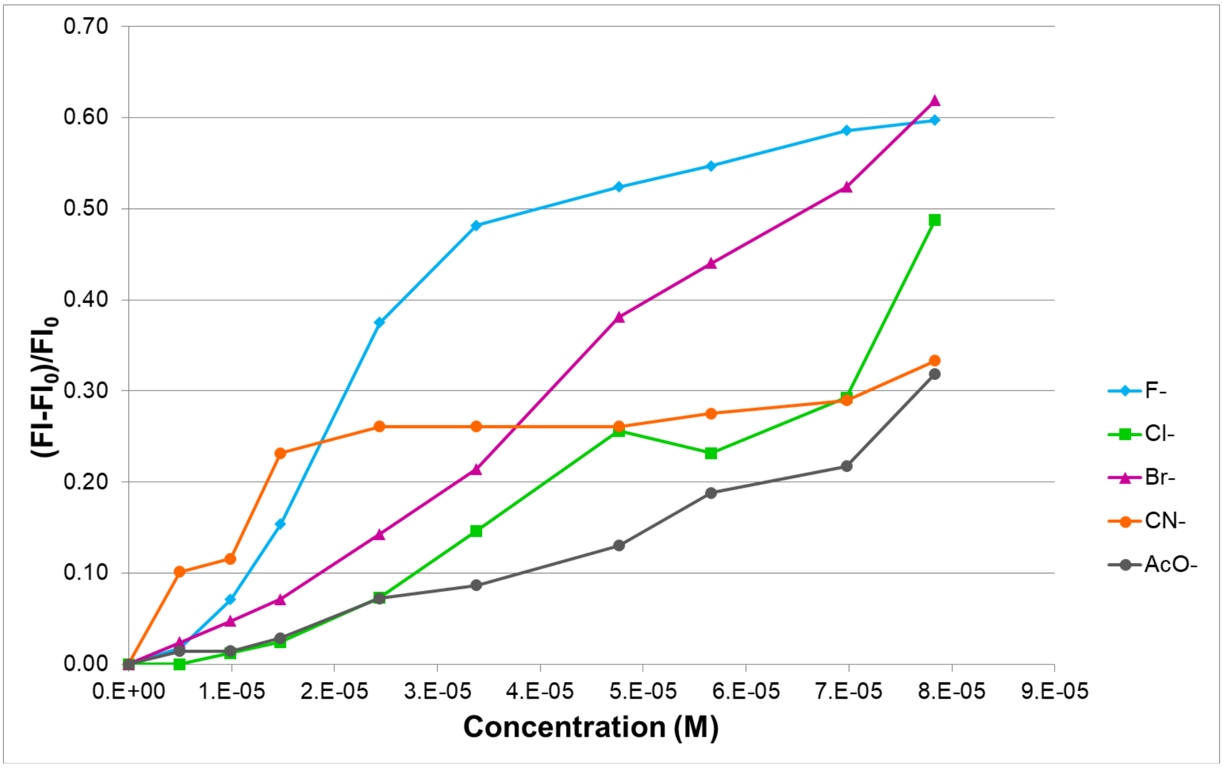

Figure S8. Fluorescence enhancement factor (FI-FI$_0$/ FI$_0$) obtained in the titration of compound **2c** with increasing concentrations of different anions in acetone.
FI: Fluorescence emission intensity value for the examined concentration of anion.
FI$_0$: Fluorescence emission intensity value obtained for the sensor in absence of anion.

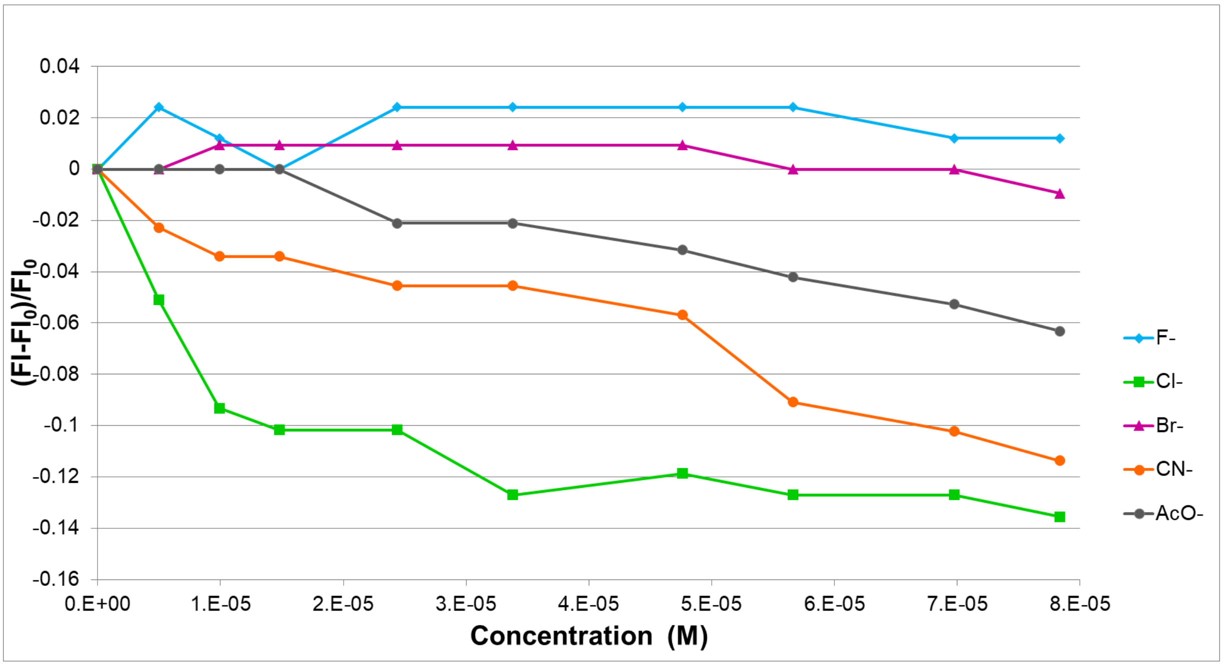

Figure S9. Fluorescence enhancement factor (FI-FI$_0$/ FI$_0$) obtained in the titration of compound **2c** with increasing concentrations of halide anions in ethanol.
FI: Fluorescence emission intensity value for the examined concentration of anion.
FI$_0$: Fluorescence emission intensity value obtained for the sensor in absence of anion.

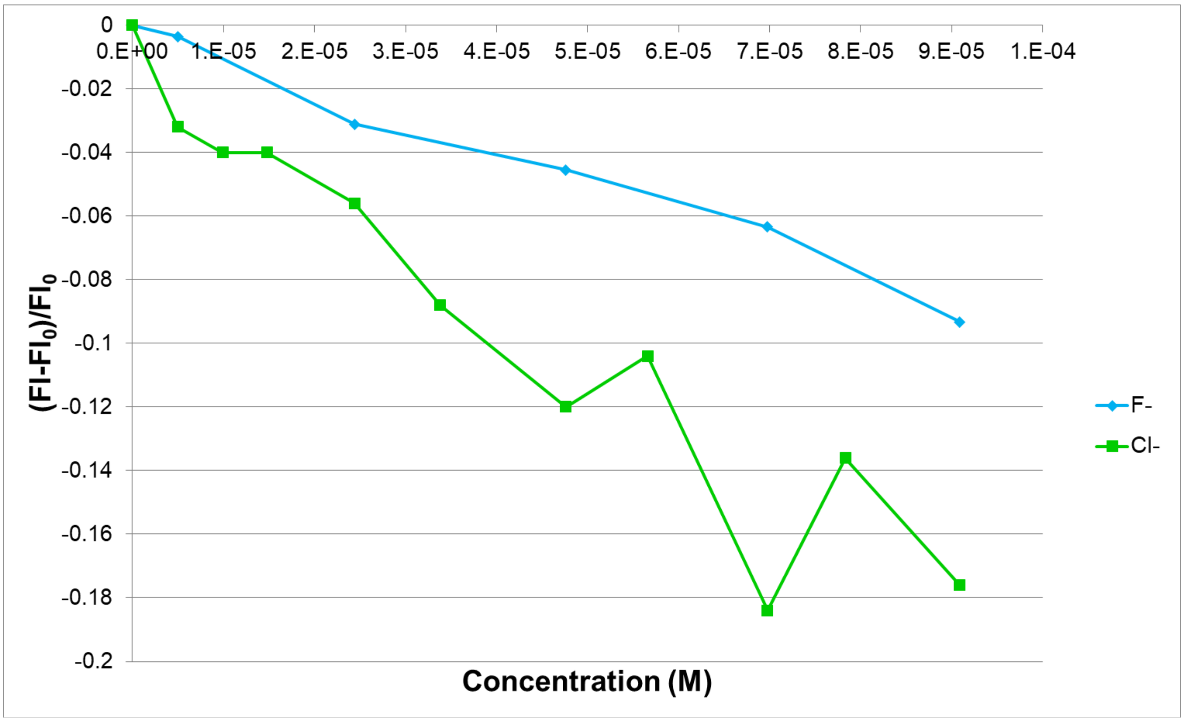

Figure S10. Fluorescence enhancement factor ($FI\text{-}FI_0$/ FI_0) obtained in the titration of compound **2e** with increasing concentrations of different anions in acetone.

FI: Fluorescence emission intensity value for the examined concentration of anion.

FI_0: Fluorescence emission intensity value obtained for the sensor in absence of anion.

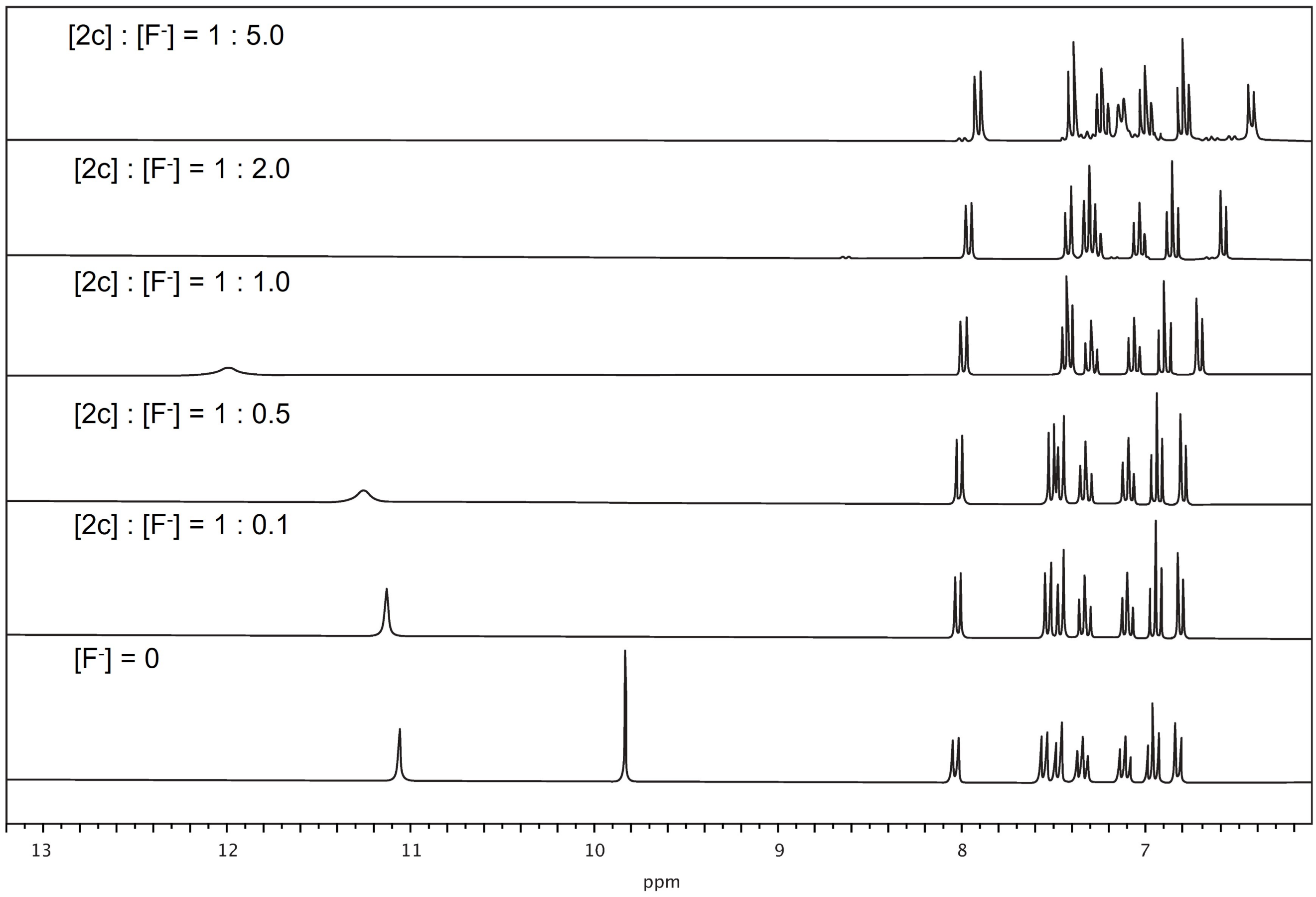

Figure S11. ^{1}H-NMR spectra of fluorescence sensor **2c** in d_6-DMSO in the presence of fluoride anion at molar ratio indicated over each spectrum.

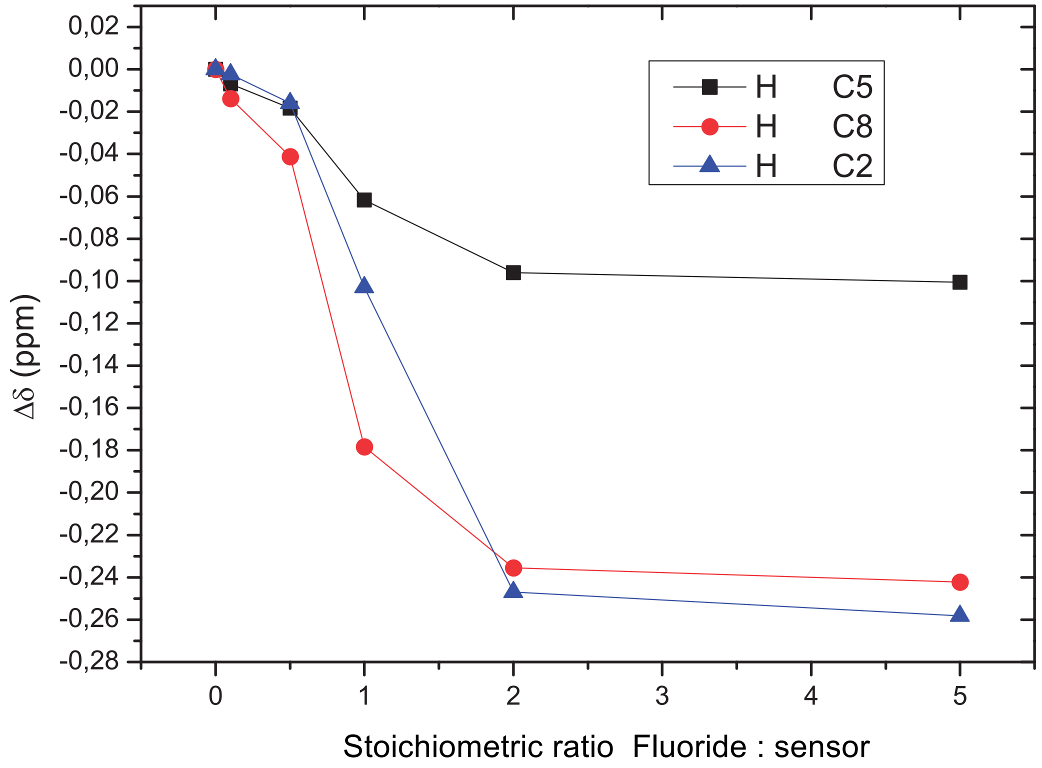

Figure S12. Decrease in the ^{1}H-NMR spectra of fluorescence sensor **2c** in d_6-DMSO in the presence of fluoride anion at molar ratio indicated over each spectrum.

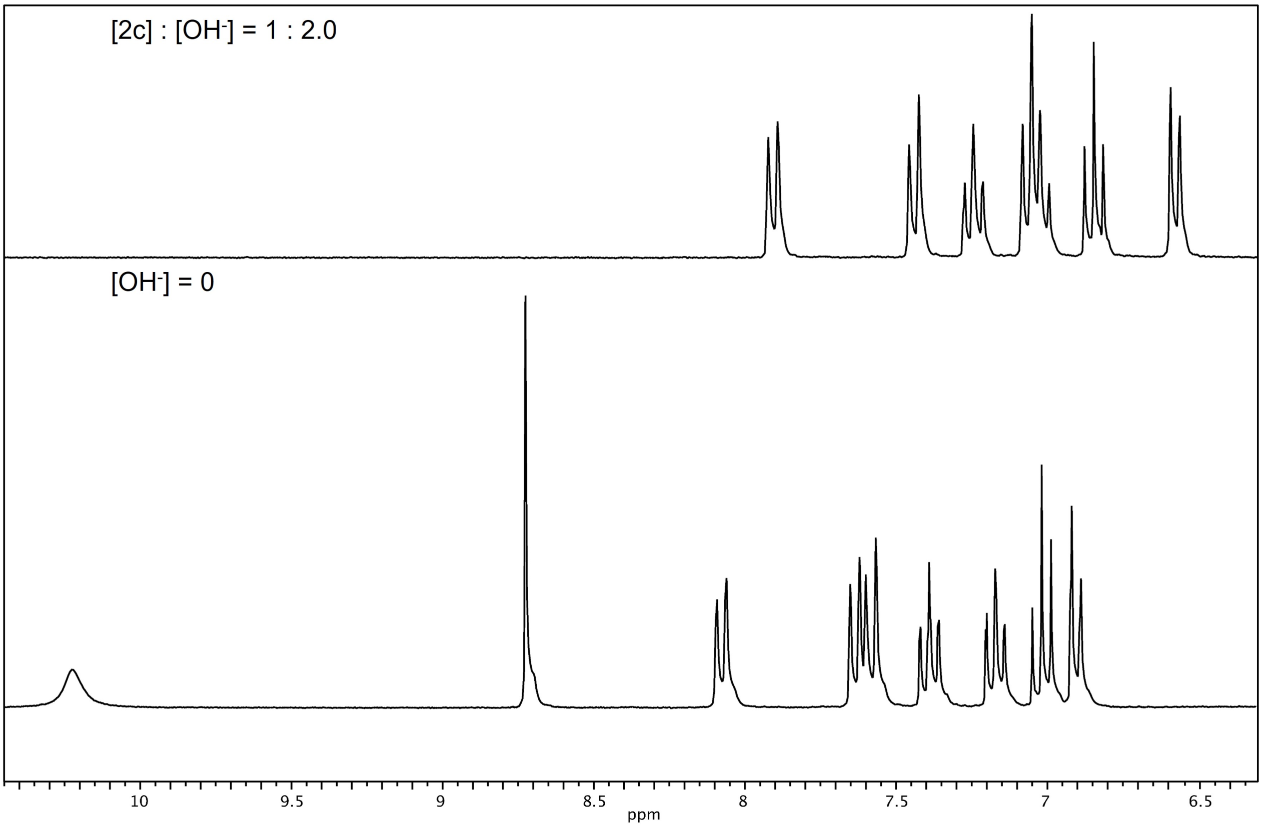

Figure S13. ^{1}H-NMR spectra of fluorescence sensor **2c** in d_6-acetone in the presence of hydroxide anion at molar ratio indicated over each spectrum.

Table S1**.** Solvatochromic effect. Fluorescence emission maxima and intensities (in parenthesis) of compounds **2c** and **2e** in solvents with different polarities.

**Solvent**	ε	**2c**		**2e**	
		λ_{exc}[1]	λ_{em}[2] (FI[3])	λ_{exc}[1]	λ_{em}[2] (FI[3])
Hexane	1.9	250, 290*, 320, 334	318, 342 (47.2), 356, 366	256, 302*, 334	332, 346 (10.4)
Cyclohexane	2.02	252, 288*, 322, 334	320, 342, 356 (72.3)	256, 302*, 334	332, 346 (34.2)
Dioxane	2.21	250, 290*, 324, 338	320, 352 (64.5), 366	260, 306*, 334	342, 352 (27.3)
Benzene	2.3	290*, 324, 336	348 (46.3), 362	306*, 334	336, 350 (22.5)
1-propanol	20.1	260, 290, 338*	380 (15.2)	258, 304*, 336	340, 354 (93.9)
Ethanol	24.3	252, 290*, 326, 348, 356	320, 370 (35.9)	260, 304*, 334	340, 354 (87.9)
Acetone	20,7	338*	364 (42.4)	329*	348 (100), 364
Methanol	33.6	250, 290*, 306	316, 370 (39.0)	260, 304*, 336	340, 354 (83.6)
Acetonitrile	38.3	250, 290*, 324, 336	320, 366 (46.8)	260, 304*, 336	340, 352 (86.2)
DMSO	45.0	332*, 340	366, 374 (33.8)	310*, 334	342, 358 (85.7)
Water	80.3	254, 290*, 332	324, 368 (6.9)	258, 302*, 336	342, 354 (99.1)

$\boldsymbol{\varepsilon}$:Dielectric Constant. [1]excitation wavelength in nm (*λ_{exc} employed for obtaining the fluorescence emission spectra), [2]emission wavelength in nm, [3]FI: fluorescence intensity, in parenthesis, at the maximum emission wavelength. The fluorescence intensity is referred to the most intensity fluorescence value obtained and assigned as 100.

Table S2. Affinity constant values obtained for fluoride with sensor **2c** and chloride with sensor **2c**.

**Anion**	**Slope**	**Ordinate**	R^2	K_{ass}	**log** K_{ass}
F^-	8.49×10^{-8}	1.32×10^{-2}	0,9794	1.55×10^{5}	5.19
Cl^-	2.95×10^{-7}	2.85×10^{-2}	0,9242	9.64×10^{4}	4.98

The association constants sensor-anions, K_{ass}, were determined using the Benesi- Hildebrand treatment. The double reciprocal plots were obtained by correlation 1/FI-FI_0 (FI is the fluorescence intensity in arbitrary units for a value of anion concentration and FI_0 is the fluorescence intensity obtained for the sensor in absence of anion. K_{ass} can be calculated by simply dividing intercept by the slope according to the following equation:

$$K_{ass} = \frac{[CZ\text{- -}A^-]}{[CZ][A^-]}$$

$$\frac{1}{F} = \frac{1}{K_{ass}k\phi}\frac{1}{c_{CZ}}\frac{1}{c_{A^-}} + \frac{1}{k\phi}\frac{1}{c_{CZ}}$$

Where:
F is the fluorescence intensity or the increase in the fluorescence intensity (FI-FI_0)
K_{ass} is the affinity or association constant
k is an instrumental constant
Φ is the fluorescence quantum yield of the complex sensor-anion
C_{CZ} is the sensor concentration (constant)
C_{A-} is the anion concentration (variable)

This treatment is described in Martn, L.; Len, A.; Olives, A.I.; del Castillo, B.; Martin, M.A. Spectrofluorimetric determination of stoichiometry and association constants of the complexes of harmane and harmine with beta-cyclodextrin and chemically modified beta-cyclodextrins, *Talanta* **2003**, *60*, 493-503.

Table S3**.** Radius predicted for the anions to be recognized by the sensor **2c**.

Sensor or anion	Radius (Å)
**2c** cavity	2.35[1]
F^-	1.19
Cl^-	1.72
Br^{--}	1.88
CN^-	1.91

[1] This value corresponds to the distance between the NH and the OH group hydrogens.

Table S4**.** Linear regression parameters obtained for the anions studied and the sensor **2c** in acetone.

Anion	slope	ordinate	R^2
F^-	1.40×10^5	14.54	0.9702
Cl^-	5.90×10^4	7.61	0.9355
Br^{--}	6.80×10^4	8.15	0.9957
CN^-	2.00×10^4	7.67	0.8508
AcO^-	6.10×10^4	6.00	0.7440
OH^-	6.70×10^3	1.79	0.8728

Table S5. Determination of fluoride and chloride anions in real samples. Accuracy evaluation.

Anion	Real sample	Relative error (%)
F^-	Mouthwash solution“Fluor Kin”	3.5
Cl^-	medicinal spring water	5.1
